# Africa in the Time of Cholera: A History of Pandemics from 1817 to the Present

**DOI:** 10.3201/eid1802.111535

**Published:** 2012-02

**Authors:** Iruka N. Okeke

**Affiliations:** Haverford College, Haverford, Pennsylvania, USA

**Keywords:** Africa, cholera, historical, pandemics, book review

In contrast to the setting of Love in the Time of Cholera by Gabriel Garcia Márquez, we live in a time when no one should have to contract or die of cholera. Nonetheless, ≈100,000 persons in Africa contracted cholera in 2011 alone, and >2,500 died in what Mintz and Guerrant have referred to as an “unconscionable tragedy” (*1*). In Africa in the Time of Cholera, Echenberg chronicles how, within a century, cholera has been transformed from an imported scourge to an African disease.

Echenberg sets the stage with a concise historical overview, depicting the eventual triumph over cholera in most of the world as a technological conquest over the original technological advances that created pandemic cholera. The first part of the book, devoted to the first 6 pandemics, is a lucid account of cholera in Africa before the 1950s. It includes some lesser known facets of cholera history, such as James Christie’s excellent mid-19th century epidemiologic work in Zanzibar and the political consequences of that period’s cholera outbreaks in Tunisia.

The second part, which covers the current and 7th cholera pandemic, begins with an overview of medical advances that have yielded today’s intervention tools. The section unfortunately includes a smattering of scientific inaccuracies, mostly related to the cell biology of *Vibrio cholerae*, in an otherwise well-researched and accessible book. Drawing from biomedical as well as historical sources, Echenberg demonstrates how the failure to provide clean water and sanitation to most of Africa’s inhabitants has led to explosive human and financial costs from cholera. He sketches portraits of these failures in countries with different histories and governance problems, illustrating infrastructural setbacks that have enabled cholera to erupt in present day Angola, South Africa, Senegal, and Zimbabwe.

The book highlights the success of oral rehydration therapy for cholera case management, and the futility of antimicrobial drug strategies because of drug resistance. Echenberg touches on the potential for vaccines, with appropriate caution, emphasizing that vaccination is no substitute for plumbing. Unfortunately, vaccines are often presented in a manner that entangles their weaknesses with those of antimicrobial drugs, underplaying the potential advantages that vaccines may have over drugs in dealing with outbreaks once they occur. It has taken cholera experts decades to advocate for vaccine use in high risk settings, culminating in World Health Assembly resolution WHA64.15 in May 2011, which urges that all states “give consideration to the administration of vaccines, where appropriate, in conjunction with other recommended prevention and control methods and not as a substitute for such methods.”

The most compelling arguments for vaccine use in conjunction with preventive interventions were published just as the book was being completed (*2*), and it is unfortunate that they were not included in this otherwise commendable analysis of intervention possibilities. However, Echenberg is to be commended for the strength of the key message of the book: that lack of potable water and sanitation, the factors that eliminated cholera from much of the world, is the principal reason why today’s cholera crisis (excluding complex emergencies, perhaps typified by the ongoing epidemic in Haiti) is largely African.

The story of cholera in Africa is long overdue and timely. In his usual engaging and accessible style, Echenberg has written another book that infectious disease experts should read for historical and social perspectives on the diseases they investigate and treat.
